# Transcription factor Sp4 is required for hyperalgesic state persistence

**DOI:** 10.1371/journal.pone.0211349

**Published:** 2019-02-27

**Authors:** Kayla Sheehan, Jessica Lee, Jillian Chong, Kathryn Zavala, Manohar Sharma, Sjaak Philipsen, Tomoyuki Maruyama, Zheyun Xu, Zhonghui Guan, Helge Eilers, Tomoyuki Kawamata, Mark Schumacher

**Affiliations:** 1 Department of Anesthesia and Perioperative Care, University of California, San Francisco, San Francisco, California, United States of America; 2 Department of Cell Biology, Erasmus University Medical Center, Rotterdam, The Netherlands; 3 Department of Anesthesiology, Wakayama Medical University, Wakayama, Japan; Indiana University School of Medicine, UNITED STATES

## Abstract

Understanding how painful hypersensitive states develop and persist beyond the initial hours to days is critically important in the effort to devise strategies to prevent and/or reverse chronic painful states. Changes in nociceptor transcription can alter the abundance of nociceptive signaling elements, resulting in longer-term change in nociceptor phenotype. As a result, sensitized nociceptive signaling can be further amplified and nocifensive behaviors sustained for weeks to months. Building on our previous finding that transcription factor Sp4 positively regulates the expression of the pain transducing channel TRPV1 in Dorsal Root Ganglion (DRG) neurons, we sought to determine if Sp4 serves a broader role in the development and persistence of hypersensitive states in mice. We observed that more than 90% of Sp4 staining DRG neurons were small to medium sized, primarily unmyelinated (NF200 neg) and the majority co-expressed nociceptor markers TRPV1 and/or isolectin B4 (IB4). Genetically modified mice (Sp4+/-) with a 50% reduction of Sp4 showed a reduction in DRG TRPV1 mRNA and neuronal responses to the TRPV1 agonist—capsaicin. Importantly, Sp4+/- mice failed to develop persistent inflammatory thermal hyperalgesia, showing a reversal to control values after 6 hours. Despite a reversal of inflammatory thermal hyperalgesia, there was no difference in CFA-induced hindpaw swelling between CFA Sp4+/- and CFA wild type mice. Similarly, Sp4+/- mice failed to develop persistent mechanical hypersensitivity to hind-paw injection of NGF. Although Sp4+/- mice developed hypersensitivity to traumatic nerve injury, Sp4+/- mice failed to develop persistent cold or mechanical hypersensitivity to the platinum-based chemotherapeutic agent oxaliplatin, a non-traumatic model of neuropathic pain. Overall, Sp4+/- mice displayed a remarkable ability to reverse the development of multiple models of persistent inflammatory and neuropathic hypersensitivity. This suggests that Sp4 functions as a critical control point for a network of genes that conspire in the persistence of painful hypersensitive states.

## Introduction

Pain arising from peripheral tissue and/or nerve injury is driven by activity in nociceptors [1–3]. Depending on the inciting event (inflammation, nerve injury), not only peripheral but also spinal and/or supraspinal signaling pathways can all conspire to amplify and produce persistence of pain [4–6]. At the level of the nociceptor, the basis of acute inflammatory pain and its persistence has been studied with a focus on inflammation-induced modifications of ion channel function that result in lowering activation thresholds in the presence of the ongoing production of endogenous sensitizing molecules [3, 7, 8]. However, other processes that drive the persistence and transition from acute to chronic pain continue to be examined [5, 9–12].

Inflammation and/or neuropathy-induced changes in nociceptor gene expression have also been proposed as a driver in pain persistence. For example, studies linking an increase in the expression of TRP channels support tissue-injury induced changes in nociceptor transcription of TRPV1 to profoundly affect nociceptor signaling [13–15]. We have previously characterized transcriptional control elements responsible for the expression of TRPV1 in nociceptors [16, 17]. This analysis revealed a TRPV1 dual promoter system (P1 and P2) that is positively regulated by Nerve Growth Factor (NGF) [17]. The proximal, P2 promoter contains a GC-rich DNA binding domain that is required for TRPV1 transcriptional activity. Two members of the Sp1-like transcription factor family, Sp4 and to a lesser extent Sp1, bind to the TRPV1 P2 promoter domain and are proposed to positively regulate TRPV1 expression [18].

Sp4 is a member of the Sp1-like transcription factor family and is predominantly expressed in neurons [19–22]. Sp4 has been linked to various neuronal processes including signaling [23–26], energy production [27, 28] and conditions such as bipolar disorder [29–31]. Members of the Sp1-like transcription factor family are distinguished by their ability to bind GC–box domains, which are often associated with a gene’s upstream promoter region. Although Sp1-like members share certain common characteristics of binding to GC-rich targets *in vitro*, they display remarkable diversity for gene-specific regulation *in vivo* [32–34]. Given that TRPV1 is necessary for the development of inflammatory thermal hyperalgesia and is implicated in other experimental and clinical pain states [11, 35–39], we sought to understand the role of Sp4 in nociception, *in vivo*. Although Sp4 is known to be expressed in the central nervous system [40–43], we now establish the pattern of expression of Sp4 in Dorsal Root Ganglion (DRG) and investigate its role in models of inflammatory and neuropathic pain. We propose that transcription factor Sp4 is required for the persistence of pain states driven by inflammatory and neuropathic conditions.

## Materials and methods

### Mice

Sp4+/- C57Bl/6 heterozygous and Wild type (wt.) mice were a gift from the Department of Cell Biology, Erasmus MC, Rotterdam, The Netherlands, and genotype was confirmed [44]. Behavioral testing of Sp4+/- heterozygous mice with a 50% reduction in Sp4 was conducted because prior study of mice with marked attenuation of Sp4 (2–5% of residual activity) showed structural brain defects [45], and mice with a homozygous deletion of the Sp4 N-terminal activation domain appear normal at birth but the majority died by 1 month of age [19, 44]. Sp4+/- mice develop normally and are indistinguishable from their wt. litter mates in terms of baseline thermal, mechanical or cold threshold testing, baseline weight or spontaneous activity as measured by video tracking (Bioseb). TRPV1-/-; TRPV1 +/- heterozygous, and C57Bl/6 mice were obtained from the Jackson Laboratory: Bar Harbor, ME and genotype confirmed. Mice weighing 25–30 g were housed in a climate-controlled room on a 12 hour light and 12 hour dark cycle. A lab diet was available *ad libitum*, with the exception of when the mice were being tested. Separate groups of male mice were used for mechanical, thermal and cold sensitivity behavioral testing due to apparent cross testing learning. Efforts were made to minimize the number of mice used and their discomfort. Experimental protocols were approved by the University of California, San Francisco, Institutional Animal Care and Use Committee (IACUC).

### Testing conditions

Freund’s Complete Adjuvant (CFA) [46] 20 μl emulsified with saline or alternately saline alone as vehicle control were injected into the left hindpaw plantar surface of mice. Nerve Growth Factor (NGF): 20μl of hNGF (Invitrogen) Life technologies: Recombinant Human Protein 11050-HNAC-50) (4μg / 20ul / mouse) versus saline vehicle control was intraplantarly (ipl) injected into the left hindpaw’s plantar surface [47]. A Spared Nerve Injury (SNI) model of neuropathic pain versus sham control was performed in mice through the ligation and transection of the sural and superficial peroneal branches of the sciatic nerve, leaving the tibial nerve intact as described [48, 49]. Oxaliplatin-based model of neuropathic pain was accomplished by injection of mice with oxaliplatin (Sigma) intraperitoneal (ip) 3mg/kg in saline versus saline alone vehicle control as described [50].

### Behavioral testing

#### Thermal latency (hargreaves test)

Mice were acclimated and baseline measurements were taken 3 days, 2 days, and one hour before injection. Mice were placed in translucent chambers on a glass plate. Heat sensitivity was measured using an infrared source aimed at the plantar surface of the left hindpaw [51]. Paw withdrawal latencies were measured, with a maximum cut-off time of 20 seconds (to avoid tissue damage). Measurements were taken three times per testing point, with at least 5 minutes of rest between tests.

#### Mechanical (von Frey)

Mechanical sensitivity was quantified as a paw withdrawal threshold in response to calibrated monofilament increasing strength. Mice were placed in plastic cages on a wire net platform. A series of calibrated von Frey filaments (starting with .4 g) were applied perpendicularly to the plantar surface of the hindpaw with enough force to bend the filament for approximately 1 second. This was done three times, with ten seconds between each testing. Paw flinch and/or withdrawal were considered a positive response. The strength of the filament was increased or decreased following a negative or positive response (respectively). The stimulus producing a 50% likelihood of withdrawal response was calculated using the “up-down” method [52]. This procedure was applied 4 times following the first change in response. This measurement was taken three times per mouse, with 15–20 minutes of rest between each testing.

#### Cold plate

Following acclimation, mice were tested at 10°C and following a 20 minute rest interval, again at 4 ^o^C, for a maximal observation period of 20 sec / trial using a Cold Plate (Bioseb—Cold Plate, USA) and translucent chamber. The time (seconds) until the first paw flinch / shake was observed was recorded as previously described [50, 53].

### Immunohistochemistry and quantitative image analysis

Male C57Bl/6 mice were sacrificed per UCSF IACUC protocol and the left L4 and L5 DRGs removed and immersed in 4% paraformaldehyde in 0.1-M phosphate buffer (PB) for 3 hours at 4°C and then cryoprotected in 25% sucrose in 0.01-M phosphate-buffered saline (PBS) overnight at 4°C. The samples were then placed in TissueTek embedding medium (Sakura, Tokyo, Japan) and rapidly frozen. 12 μm sections were cut using a sliding cryostat (LEICA, Tokyo, Japan). Tissue sections were thaw-mounted onto gelatin-coated slides, washed in 0.01-M PBS for 30 minutes followed by 0.2% Triton X-100 (Sigma) in 0.01-M phosphate-buffered saline (PBS-t) for 30 minutes and incubated for 60 minutes at room temperature in a blocking solution consisting of 1% normal donkey serum and 0.01-M PBS-t. The sections were then incubated with a mixture of the primary antibodies in the blocking solution overnight at 4°C. After rinses with 0.01-M PBS-t, slices were incubated with Alexa Fluor 488-, Alexa 597-, and Alexa 649-labeled species-specific secondary antibodies (Invitrogen, Carlsbad, CA) at a dilution of 1:500 in PBS-t for 2 hours at room temperature. Images for quantitative analysis were acquired using an epifluorescence microscope (Axiovert 200, Carl Zeiss) and for representative data using a confocal laser scanning microscope (ECLIPSE C1, Nikon, Tokyo, Japan).

Antibodies: Rabbit anti-Sp4 (1:1000, SC-645, Santa Cruz), goat anti-TRPV1 (1:200, SC-12498, Santa Cruz), mouse anti-neurofilament 200kD (NF200; 1:4000, N0142, Sigma), goat anti-peripherin (1:200, SC-7604, Santa Cruz). Mouse anti-protein gene product 9.5 (PGP9.5; 1:2000, MO25010, Neuromics). Biotinylated isolectin B4 (IB4; 1:100, L3759, Sigma). Previous studies have confirmed the specificity of anti-Sp4 antibody [43] anti-NF200 and anti-peripherin antibody [54] used in this study. In addition, preliminary studies confirmed the specificity of the Sp4 antibody based on its retinal neuronal staining in mice [43] and the anti-TRPV1 antibody using TRPV1-deficient mice.

Images taken with epifluorescence microscope were imported into Image J software (NIH, Bethesda, MD) for quantitative analysis and Sp4 staining quantified using gray scales (0-black to 255-white). For Sp4 immunoreactivity, DRG cells exhibiting intense nuclear staining with a grayscale 2.0-times greater than that of the background was considered as Sp4-positive. The cross-sectional area of DRG neuronal cell bodies were visualized by counterstaining with a pan-neuronal marker, PGP9.5 and measured using AxioVision 4.8 (Carl Zeiss). For colocalization of Sp4 with other neuronal markers, only neurons with clearly visible nuclei were counted in each case. Quantitative analyses were performed on four randomly selected sections from each DRG of 4 mice. Because a stereological approach was not used, quantification of data may have yielded biased estimates of actual numbers of cells. To prevent duplicate counting, we used only sections that were at least 48 μm apart.

### Cell culture

Primary DRG neuronal cultures were derived from 6 to 8 week-old Sp4+/- and wt. mice using previous methods described [55, 56].

### Calcium imaging

Measurement of [Ca^++^]_I_ changes in primary DRG neurons derived from Sp4+/- or wt. mice was accomplished by plating DRG neurons on coverslips coated with poly-DL-ornithine / laminin and preloaded with the calcium dye, Fura-2 AM (Invitrogen, Carlsbad, CA), for 40 min at 37°C (2.5 μM Fura-2 in HBSS + 20 mM HEPES + 0.1% BSA). DRG neurons were visualized through a motorized Axiovert 200 microscope (Carl Zeiss Light Microscopy, Germany) equipped with an ICCD camera (Stanford Photonics, Stanford, CA) and controlled through the Imaging Workbench software package (Indec Biosystems, Mountain View, CA) as previously described [56].

### qRT-PCR

RNA was isolated from mouse lumbar DRG (Trizol Reagent–Invitrogen, Carlsbad, CA) and first strand cDNA was prepared (Agilent Technologies). Using the StepOnePlus Real-Time PCR system (Applied Biosystems, Carlsbad, CA), cDNA samples were probed with the following primers: (Applied Biosystems-ABI) Sp4 (Cat# Hs00162095_ml), Sp1 F: AATTTGCCTGCCCTGAGTGC; R: TTGGACCCATGCTACCTTGC [57], Sp3 F: CAGATCATTCCTGGCTCT; R: TCTAGATCGACACTATTGAT [58], custom primers (Invitrogen (Life Technologies) TRPV1 F: CCC ATT GTG CAG ATT GAG CAT; R: TTC CTG CAG AAG AGC AAG AAG C; TRPA1 F: GCA GGT GGA ACT TCA TAC CAA CT; R: CAC TTT GCG TAA GTA CCA GAG TGG; TRPM8 F: GTG TCT TCT TTA CCA GAG ACT CCA AGG CCA; R: TGC CAA TGG CCA CGA TGT TCT CTT CTG AGT; ASIC3 F: TCACCTGTCTTGGCTCCTC; R: TGACTGGGGATGGGATTTCTAAG [59]; TRPV4 F: CCTTGTTCGACTACGGCACTT; R: GGATGGGCCGATTGAAGACTT [60]; Piezo1 F: CACTCTGCAGCCACAGACAT; R: CACACATCCAGTTGGACAGG [61]; Piezo2 F: GCCCAGCAAAGCCAGCTGAA; R: GGGCTGATGGTCCACAAAGA [61]; TREK-1 F: TTTTCCTGGTGGTCGTCCTC; R: GCTGCTCCAATGCCTTGAAC [62]; TMEM150c F: GGCATGGACGGGAAGAAATGC; R: CCAAGGACAAACTGTTGCTACACC [63]. The mRNA expression levels of the genes analyzed were normalized by GAPDH expression F: TGCGACTTCAACAGCAACTC; R: CTTGCTCAGTGTCCTTGCTG and represented as Relative Quantitation (RQ) using the comparative *C*_T_ method as previously reported [18, 56, 64]

### Statistics and analysis

Mean values were expressed as +/- SEM. When applicable, detection of behavioral differences between multiple groups were by two-way RM ANOVA followed by Bonferroni post-hoc test. Differences in DRG mRNA were determined with a two tailed unpaired t-test. A minimum P value less than 0.05 was considered to show a significant difference. Analysis was performed using Prism (GraphPad Software, La Jolla, CA).

## Results

### Transcription factor Sp4 is expressed in nociceptive neurons

**Fig 1:**
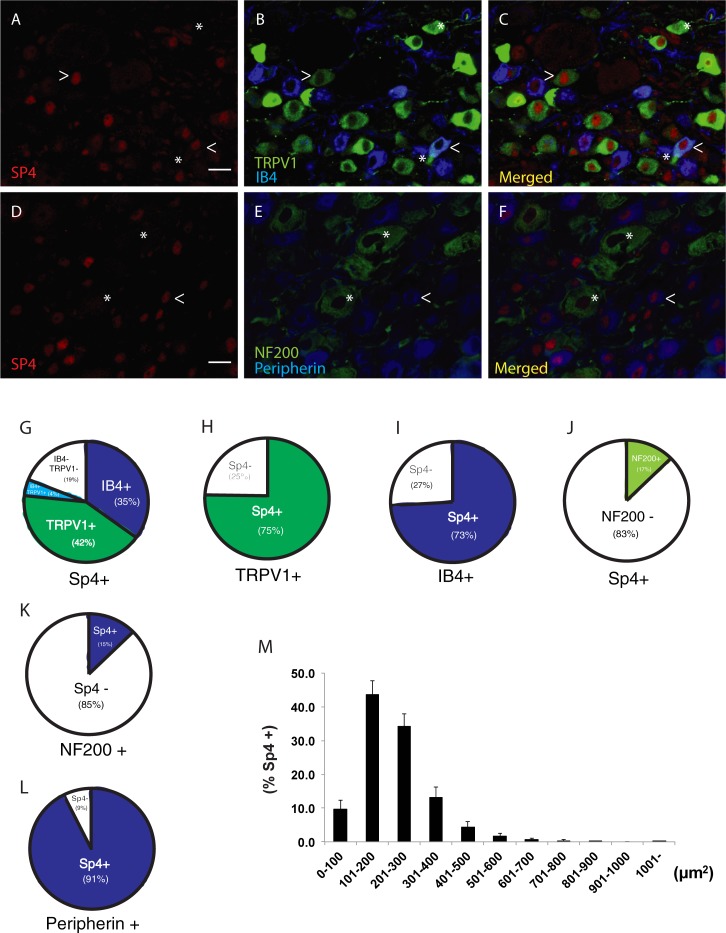
Sp4 is expressed in small to medium size DRG neurons expressing nociceptor markers. Triple immunofluorescence of **(A)** Sp4+ nuclear (red), **(B)** TRPV1+ (green) and IB4+ (blue) cytoplasmic staining, **(C)** Merged image. (>) indicates Sp4+ neurons co-staining with either TRPV1 or IB4 respectively. (*) indicates example of Sp4—neurons co-staining for TRPV1 or IB4 respectively. Triple immunofluorescence of **(D)** Sp4+ nuclear (red), **(E)** NF200 (green) and Peripherin (blue), **(F)** Merged image. (<) indicates small Sp4+, Peripherin+, NF200—neuron. (*) indicates Sp4 -, Peripherin+, NF200+ large neuron. **(G)** Pie chart illustrating percentage of Sp4+ neurons co-staining with either TRPV1+ (42% green), IB4 + (35% blue), TRPV1+ and IB4+ (4%) as compared to Sp4+, TRPV1 -, IB4 - (19%). Overall, 81% of Sp4+ DRG neurons co-expressed one or both (TRPV1, IB4) nociceptor markers with a total of 4,150 Sp4+ neurons from 4 mice analyzed. **(H)** 75% of TRPV1+ DRG neurons co-expressed Sp4 with 1,765 TRPV1+ neurons analyzed. **(I)** 73% of IB4+ DRG neurons co-expressed Sp4 with 1,466 IB4+ neurons analyzed. **(J)** Percentage of Sp4+ DRG neurons that are NF200 positive (+) or negative (-). A total of 83% of Sp4+ neurons were NF200—with a total of 3,304 Sp4+ neurons from 4 mice analyzed. **(K)** 15% of NF200+ DRG neurons were Sp4+ with a total of 2,357 NF200+ neurons counted from 4 mice. **(L)** A total of 91% of peripherin+ neurons were Sp4+ with 4,381 peripherin+ neurons counted from 4 mice. **(M)** Cell size distribution of Sp4+ DRG neurons. Each bar represents the percentage of Sp4+ neurons within each size range of cross-sectional areas among the total Sp4+ neurons. The cross-sectional area (size) frequency distribution (%) of Sp4+ neurons exhibited a major peak at 100–200 and 200–300 μm^2^, with few Sp4+ nuclei found in larger neurons. More than 90% of the Sp4+ neurons measured less than 400 μm^2^. A total of 3,785 neurons from 4 individual, C57Bl/6 mice were analyzed showing Sp4+ immune staining nuclei. Scale bar indicates 20 μm.

To further understand the role of Sp4 in pain transduction, including its regulatory role in TRPV1 gene expression, we first examined the pattern of Sp4 expression in mouse DRG neurons to determine its localization relative to nociceptor markers. As shown in (Fig 1A–1C), immune-fluorescent images of Sp4 antibody staining revealed nuclear Sp4 co-localized with TRPV1+ and IB4+ staining neurons. Additional co-localization studies were performed (Fig 1D–1F) using anti-NF200 antibodies (marker of myelinated neurons) and peripherin antibody (marker of unmyelinated neurons) [65]. The majority (81%) of Sp4+ DRG neurons are co-expressed with either TRPV1 and/or IB4 (Fig 1G). Conversely, 75% of TRPV1 staining DRG neurons (Fig 1H), stained for Sp4 and a similar percentage, (73%) of IB4+ staining DRG neurons (Fig 1I) also stained for Sp4. The vast majority of Sp4+ neurons (83%) did not co-express NF200 (Fig 1J). Conversely, 15% of NF200+ DRG neurons were Sp4+ (Fig 1K) and 91% of peripherin staining DRG neurons were Sp4+ (Fig 1L) suggesting that the majority of Sp4+ neurons were non-myelinated. Taken together with our observations (Fig 1M), that more than 90% of the Sp4+ neurons measured less than 400 μm^2^, supports the idea that transcription factor Sp4 is expressed in a subpopulation of nociceptive neurons in the DRG.

### DRG derived from Sp4 +/- mice have reduced TRPV1 mRNA and capsaicin-induced calcium responses

**Fig 2:**
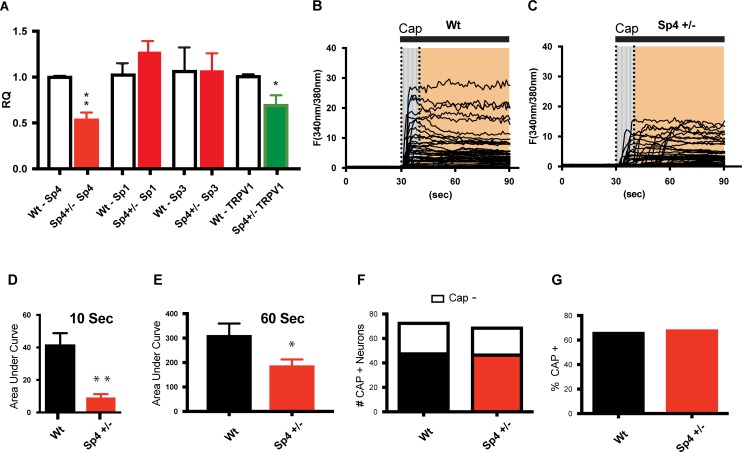
Sp4+/- DRG neurons have a reduction in TRPV1 expression and reduced capsaicin-induced calcium response. **(A)** Sp4+/- DRG expressed ~56% reduction of Sp4 mRNA. Sp1 and Sp3 DRG mRNA expression did not differ in Sp4+/- versus wt. mice (n = 6). Sp4+/- DRG expressed a ~30% reduction in TRPV1 mRNA levels as compared to wt. DRG. (* p< 0.05; ** p<0.005; triplicate samples; n = 3 independent experiments, two-way ANOVA with Bonferroni’s test). **(B)** Individual traces of Fura-2 fluorescence ratio 340nm/380nm excitation and 510nm emission in wild-type (wt) and **(C)** Sp4+/- DRG neurons demonstrating a reduction in the magnitude of the capsaicin responses and the presence of delayed-onset calcium responses in Sp4+/- neurons. (Cap) capsaicin application (60 seconds) **(D)** Sp4+/- mice exhibited a 5-fold lower response magnitude (area under the curve—AUC) during the first 10 seconds (grey) (p < 0.0001; K-W) compared to wild-type neurons. **(E)** Sp4+/- DRG neurons showed an overall reduction in the magnitude (AUC) during the 60 second recording period (grey plus orange—shaded) (* p<0.05; two-tailed t test with Welch Correction). **(F)** No difference was found in the number or **(G)** percentage of capsaicin-responsive DRG neurons between Sp4+/- and wt. mice. Capsaicin (5 μM) was applied at the 30 second timing mark. Statistical comparisons: Mean area under the curve ± SEM of 3 independent experiments yielded 48 (wt.) and 46 (Sp4+/-) capsaicin-responding neurons. (Prism, GraphPad).

To gain insight into the function of Sp4 in DRG neurons, we examined the expression of Sp4, Sp1, Sp3 and TRPV1 mRNA in DRG harvested from heterozygous Sp4+/- versus wt. mice. As shown in Fig 2A, we confirmed that Sp4 mRNA levels were reduced ~50% in Sp4+/- mouse DRG. We also determined if Sp1-like family members Sp1 and/or Sp3 underwent compensatory changes in expression as a result of Sp4+/- knockdown in DRG. This is important given that Sp3 expression is slightly increased (two-fold) in embryonic E18.5 hearts of Sp4 -/- null mice [66], Sp3 and Sp4 can compete for Sp1 activation of the ADH5/FDH minimal promoter [67] and over-expression of Sp1 or Sp3 in cultured DRG neurons increased TRPV1 promoter activity in transfected DRG neurons [18]. However, no significant change in Sp1 or Sp3 mRNA was observed in Sp4+/- DRG (Fig 2A) suggesting a lack of Sp4-dependent compensation among these Sp1-like members. Finally, TRPV1 mRNA expression was reduced by ~30% in Sp4+/- compared to wt. mice.

To quantitate the change in functional expression of TRPV1 in Sp4+/- DRG neurons, we examined whether there were any detectable changes in the capsaicin-induced response properties of cultured DRG neurons derived from Sp4+/- mice. As shown in Fig 2B and 2C following the application of a capsaicin concentration expected to induce a maximal response (5 μM) [35], a striking ~80% decrease in the magnitude (AUC) of the calcium responses was observed within the initial 10 seconds of Sp4+/- DRG neuron-derived response intervals (Fig 2D). In addition, there appeared more delayed capsaicin-induced responses observed in the Sp4+/- neurons (Fig 2C). When analyzed over the entire 60 second recording-interval (AUC), a decrease in capsaicin-evoked response magnitude (Fig 2E) was also observed. The number and percentage of capsaicin—and/or KCl—responsive DRG neurons did not differ between wt. and Sp4+/- mice (Fig 2F and 2G). Overall, we observed that a relatively modest (30%) reduction of TRPV1 mRNA expression in Sp4+/- DRG was associated with a robust decrease in capsaicin-induced calcium response profiles. Given these observations, we then sought to explore the consequence of a reduction of Sp4 on the development of pain behaviors in mice under various nociceptive models beginning with inflammatory thermal hyperalgesia–a pain behavior previously shown to be dependent on TRPV1 [36, 37].

### Inflammation failed to induce persistent thermal hyperalgesia in Sp4+/- mice

**Fig 3:**
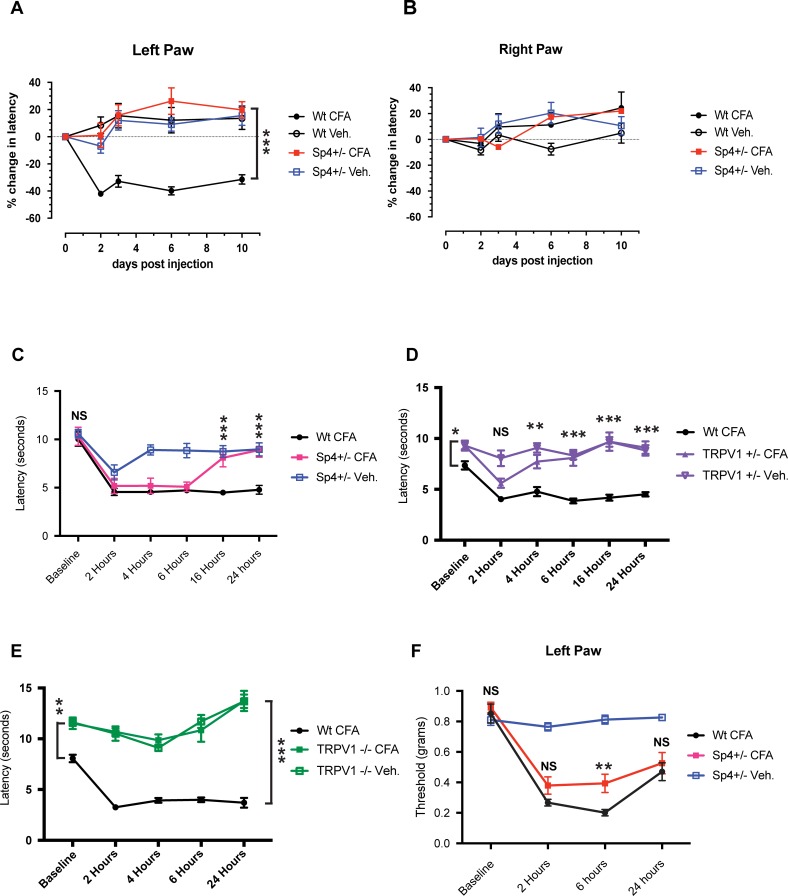
Inflammatory thermal hyperalgesia does not persist in Sp4 +/- heterozygous mice. **(A)** Percentage (%) change in left hindpaw thermal withdrawal latencies. Wild-type (wt.) mice injected with CFA (closed circles) produced a robust decrease in latency that was significantly different from Sp4+/- CFA mice on all days tested. Sp4+/- mice (closed boxes) showed no decrease when measured at days 2, 3, 6 and 10 following injection with CFA day 0. Sp4+/- CFA showed no difference compared with wt. Veh. or Sp4 Veh (*** p< 0.0001; n = 18). **(B)** Non-injected contralateral right hind-paw showed no evidence of a CFA-dependent decrease in paw withdrawal latencies (n = 18). **(C)** Thermal paw withdrawal latencies (sec) measured at baseline, 2, 4, 6, 16, and 24 hours following left hind-paw CFA injection. No differences were found between baseline thermal latency of Sp4+/- and wt mice. Following CFA paw injection, Sp4+/- mice transiently developed a decrease in paw withdrawal latency at 2, 4, and 6 hours, but showed spontaneous reversal of inflammatory thermal hyperalgesia at 16 and 24 hours (*** p<0.0001; n = 9). **(D)** Comparison of CFA-induced thermal hyperalgesia in wt. versus TRPV1+/- heterozygous mice. Baseline thermal paw withdrawal latencies of TRPV1+/- were increased compared with wt. mice (* p<0.05; n = 9). Following CFA hind-paw injection in TRPV1-/+ mice, a brief decrease in latency at 2 hours was observed that returned to TRPV1+/- baseline values and then differed from CFA—wt. latencies at 4 hours (** p < 0.0005; n = 9) and 6, 16, and 24 hours (*** p< 0.0001; n = 9). **(E)** Comparison of CFA-induced thermal hyperalgesia in wt. versus TRPV1 -/- null mice. Baseline withdrawal latencies of TRPV1-/- mice were increased compared with wt. mice (** p<0.0005; n = 6). Following CFA injection of TRPV1 -/- mice, no evidence of thermal hyperalgesia was observed and latencies differed from CFA–wt. mice for all time measured (*** p<0.0001; n = 6). **(F)** Sp4 +/- heterozygous mice developed inflammatory mechanical hypersensitivity that was diminished at 6 hours. Baseline mechanical thresholds did not differ between Sp4+/- and wt. mice. Sp4+/- CFA mice showed a diminished mechanical threshold at 6 hours when compared to wt.—CFA mice (p< 0.01; n = 12) but did not differ at 24 hours. Statistical comparisons: Bars = +/- SEM, Two-way ANOVA, with Bonferroni’s test. Prism, GraphPad).

Given our earlier observations that TRPV1 mRNA expression in primary cultured DRG neurons were positively regulated by Sp4 [18], we hypothesized that a reduction of Sp4 could disrupt the development and/or maintenance of inflammatory thermal hyperalgesia based on the notion that unencumbered Sp4-dependent transcription of TRPV1 is required for the development and/or maintenance of inflammatory hyperalgesia. Using an established model of inflammatory hyperalgesia, hind-paw injection of Complete Freund’s Adjuvant (CFA) [36], in our initial trial (Fig 3A), Sp4+/- mice failed to show development of thermal hyperalgesia on post-injection days 2, 3, 6 and 10 as compared to a robust decrease in withdrawal latency in wt. mice and its absence in the contralateral paw (Fig 3B). Nevertheless, to determine whether thermal hyperalgesia may have developed transiently in Sp4+/- mice, prior to day 2, we repeated the experiment and measured latencies at baseline, 2, 4, 6, 16 and 24 hours after CFA injection. In contrast to our initial trial, a robust decrease in paw withdrawal latency was observed at 2, 4 and 6 hours in both the Sp4+/- and wt. mice; however, this was followed by a spontaneous reversal and resolution of thermal hyperalgesia in the CFA Sp4+/- mice by 16 hours (Fig 3C).

The surprising observation that a modest baseline reduction of TRPV1 mRNA expression in Sp4+/- DRG would subsequently result in a complete loss of the persistence of inflammatory thermal hyperalgesia after 16 hours, as opposed to a diminished yet persistent thermal hyperalgesia, inspired a comparative gene-dose study with TRPV1+/- heterozygous and TRPV1-/- null mice. As compared to the equivalent baseline hind-paw withdrawal latencies measured in Sp4+/- and wt. mice (Fig 3C), baseline thermal latency values of TRPV1+/- (Fig 3D) and TRPV1-/- (Fig 3E) were greater than those observed for wt. mice. When CFA-treated TRPV1+/- mice with a 50% reduction in TRPV1 were studied, inflammatory thermal hyperalgesia was detected only at 2 hours after CFA injection, followed by a return of thermal withdrawal latencies to baseline control values by 4 hours. As previously reported, CFA-treated TRPV1-/- mice (Fig 3E) failed to develop thermal hyperalgesia throughout the 24 hour study period [36, 37]. These observations support the idea that a critical level of continually expressed TRPV1 is necessary to sustain persistent inflammatory thermal hyperalgesia.

Hind-paw injection of CFA also induces a state of mechanical hypersensitivity and/or allodynia as measured by a reduction in paw withdrawal threshold to calibrated monofilaments [52]. As shown in Fig 3F, a robust decrease in mechanical withdrawal threshold was observed following CFA injection at 2 hours in both Sp4+/- and wt. mice but with diminished mechanical hypersensitivity observed at 6 hours but not 24 hours in Sp4+/- mice. This suggests that Sp4 contributes, in part, to the maintenance of mechanical hyperalgesia. Although it is unknown which Sp4+/- dependent gene(s) are responsible for this loss of mechanical hypersensitivity, it is unlikely to be TRPV1. Despite reports of TRPV1 antagonists partially reversing cutaneous mechanical hypersensitivity [47, 68], the study of cutaneous mechanical sensitivity in models of genetic knock-down have not supported TRPV1 contributing a major role in cutaneous mechanical hypersensitive states [36, 37].

**Fig 4:**
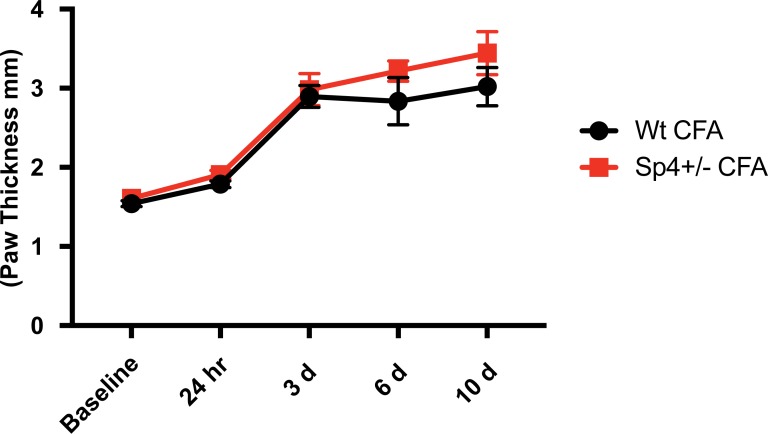
Sp4+/- did not block CFA-induced paw swelling. CFA (20 μl) emulsified with saline was injected into the left hindpaw plantar surface of wt. versus Sp4+/- mice and paw thickness (mm) measured at baseline, 24 hours, and 3, 6 and 10 days post injection. Both developed significant paw swelling over 10 days that did not differ between CFA wt and CFA Sp4+/- mice. (n = 3-6/group) Bars = +/- SEM, Two-way ANOVA, with Bonferroni’s test. Prism, GraphPad).

Given our findings that Sp4 is required for the persistence of inflammatory hyperalgesia, we investigated if this observation could be attributed to a general reduction of CFA-induced inflammation, as measured by a change in CFA-induced paw swelling [46]. As shown in Fig 4, there was no significant difference in the development or persistence of CFA-induced paw thickness between CFA Sp4+/- and CFA wt. mice throughout the 10 day study period. We interpret these results as evidence that Sp4 serves a critical role in the maintenance of inflammatory pain behaviors despite ongoing CFA-induced paw inflammation. We then sought to examine additional models of inflammatory and neuropathic pain in Sp4+/- mice including the use of a specific product of inflammation—Nerve Growth Factor (NGF).

### NGF failed to induce persistent mechanical hypersensitivity in Sp4+/- mice

**Fig 5:**
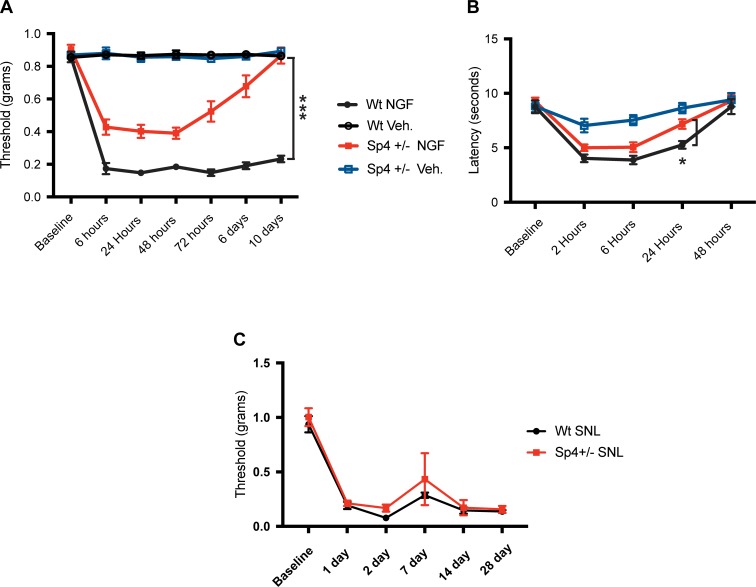
NGF failed to induce persistent mechanical allodynia in Sp4+/- mice. SNI Sp4+/- mice developed mechanical hypersenstivity. **(A)** Left hindpaw mechanical withdrawal thresholds (gms) in Sp4+/- mice following a single injection of NGF (4 ug). No significant differences in baseline mechanical thresholds were observed in Sp4+/- versus wt. mice. NGF-treated Sp4+/- mice develop a decrease in mechanical threshold at 6, 24 and 48 hours that was less than observed in NGF-treated wt. mice at 6, 24, 48, and 72 hours, and at 6 and 10 days (*** p<0.0001; n = 9). Following 48 hours, a spontaneous reversal of mechanical sensitivity was observed, with threshold withdrawal values increasing to reach baseline values by day 10 (n = 9 / group). **(B)** Thermal paw withdrawal latencies (seconds) measured at baseline and 2, 6, 24 and 48 hours following left hind-paw NGF injection. Following NGF paw injection, both Sp4+/- and wt. mice developed a transient decrease in thermal paw withdrawal latency at 2, 6 and 24 hours that returned to baseline at 48 hours. A small difference in thermal latency was found between NGF-treated Sp4+/- and NGF-treated wt. mice at 24 hours (* p<0.05; n = 9). **(C)** Following SNI nerve injury, both Sp4+/- and wt. mice developed mechanical hypersensitivity that did not differ between groups throughout the 28 day study period (n = 4 / group). Statistical comparisons: Bars = +/- SEM. Two-way ANOVA, with Bonferroni’s test. Prism, GraphPad).

NGF has been identified as a critical tissue-derived mediator of inflammatory pain capable of driving both acute and persistent pain as well as hyperalgesic states [69]. Elevated levels of NGF following tissue injury have been shown to drive pain through neuroplastic changes in nociceptor physiology and gene expression [14, 17, 70–72]. In some behavioral studies, responses to NGF paw injection also have similarities to those reported in experimental models of nerve injury-induced neuropathic pain [69]. As shown in Fig 5A, NGF induced a state of mechanical hypersensitivity for the initial 48 hours in Sp4+/- mice but one that was significantly diminished in magnitude as compared to NGF-injected wt. mice. In addition, beginning at 72 hours, the mechanical thresholds of NGF-treated Sp4+/- mice progressively returned to baseline values by day 10, becoming indistinguishable from vehicle injected wt. or Sp4+/- mice. By comparison, NGF-injected wt. mice continued to maintain a robust decrease in mechanical threshold at day 10. We also examined the effect of Sp4 on the development of NGF-induced thermal hyperalgesia. As shown in Fig 5B, NGF produced a transient decrease in thermal paw withdrawal latency over a 48 hr period. Sp4+/- initially developed thermal hyperalgesia up to 6 hours but then showed a significant reversal at 24 hrs, a profile somewhat similar to that observed with CFA Sp4+/- mice (Fig 3C). Overall, Sp4+/- mice were associated with a failure to develop persistent NGF-induced mechanical hypersensitivity and NGF-induced thermal hyperalgesia.

### Sp4+/- did not block mechanical hypersensitivity in a model of Spared Nerve injury (SNI)

Despite observations that each model of nerve injury may result in some component of inflammation [73], experimentally they are often considered distinct from primary inflammatory stimuli such as peripheral injection of CFA (Fig 3) or the isolated application of products of inflammation, e.g. NGF (Fig 5A and 5B). Considering the loss of persistent CFA and NGF-induced thermal and mechanical hypersensitivity observed in Sp4+/- mice were associated with inflammatory models, we questioned whether the SNI model would also develop persistent mechanical hypersensitivity [48, 74]. As shown in Fig 5C, following SNI, both Sp4+/- and wt. mice developed a rapid onset (1 day) and persistent (28 day) decrease in mechanical withdrawal thresholds throughout the study period. No differences in threshold testing were detected between Sp4+/- and wt. groups.

### Oxaliplatin failed to induce persistent cold or mechanical hypersensitivity in Sp4+/- mice

Although the pain associated with the platinum-based anticancer agent oxaliplatin is one of the most recognized adverse consequences of its administration, the mechanism(s) driving the associated cold allodynia and mechanical hypersensitivity remains elusive [75–77]. Rodent models using oxaliplatin have been established that emulate many of the painful symptoms experienced clinically by patients receiving oxaliplatin [78–80].These include oxaliplatin-induced decrease in cold plate latencies and cutaneous mechanical thresholds [80–83]. In contrast, persistent thermal hyperalgesia does not apparently develop in mice treated with oxaliplatin but is observed with cisplatin treatment [81]. Oxaliplatin has been shown to direct its painful hyperalgesic behaviors through a subset of specialized primary afferent nociceptors within sensory ganglion that express the cell surface lectin IB4 [80]. Since we observed 35% of Sp4+ DRG neurons co-express IB4 (Fig 1), we examined the consequence of Sp4+/- reduction on oxaliplatin-induced cold and mechanical allodynia.

**Fig 6:**
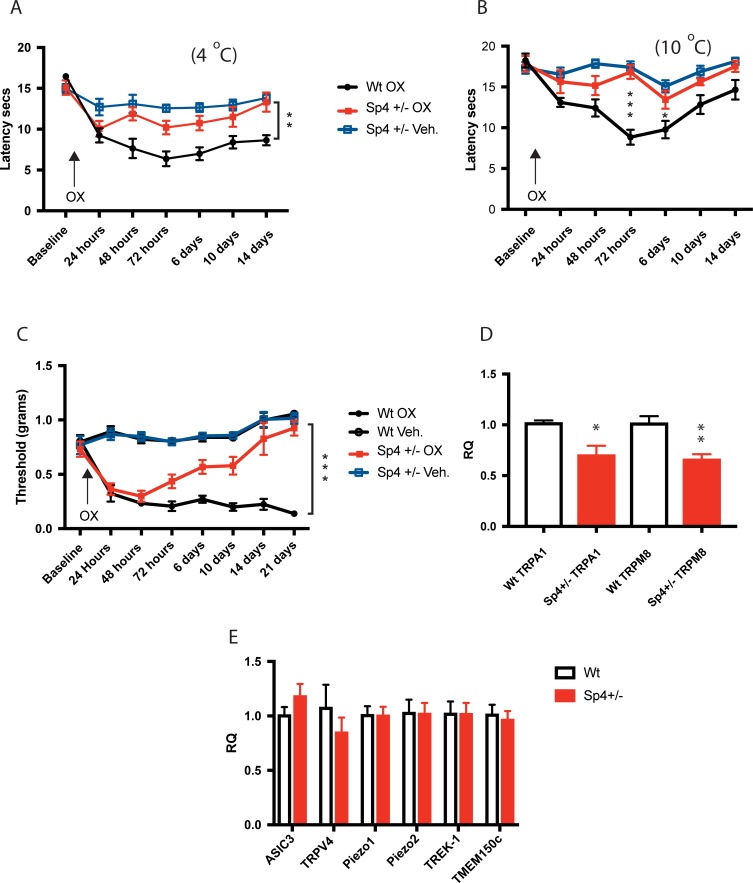
Oxaliplatin failed to induce persistent cold or mechanical hypersensitivity in Sp4+/- mice. **(A)** Sp4+/- mice injected with oxaliplatin (OX) (3mg/kg ip) on day 0, induced a decrease in 4°C cold plate withdrawal latency at 24 hours in Sp4+/- and wt. mice. Beginning at 48 hours (p< 0.005) OX-injected Sp4+/- latencies differed from OX-injected wt. mice at 72 hours and 6 days (p<0.01), 10 days (p<0.05) and 14 days (** p<0.001) with Sp4+/- OX latency values indistinguishable from Veh.–injected Sp4+/-controls at 14 days (n = 6 /group). **(B)** Cold plate testing at 10°C with differences between Sp4+/- OX and wt. OX at 72 hours (*** p<0.0001) and 6 days (* p<0.05). Sp4+/- OX and Sp4+/- Veh. did not significantly differ at 72 hours, 6 days,10 days and 14 days (N = 6 /group). **(C)** Mechanical threshold testing of Sp4+/- mice injected with OX (3mg/kg ip) on day 0, induced a decrease in mechanical threshold within 24 hours in both Sp4+/- and wt. mice. Beginning at 72 hours (p<0.05), Sp4+/- OX induced mechanical allodynia progressively reversed at 6 days (p<0.005), 10 days, 14 days and 21 days (*** p<0.0001) approaching Veh—injected control values by 14–21 days. wt. OX mice showed mechanical allodynia days 1–21 (n = 6). Two-way RM ANOVA, with Bonferroni’s test. **(D)** Sp4+/- DRG expressed a reduction in TRPA1 and TRPM8 mRNA levels as compared to wt. DRG. **(E)** No change in ASIC3, TRPV4, Piezo1, Piezo2, TREK-1 or TMEM150c mRNA expression was detected in Sp4+/- as compared to wt. DRG (* p< 0.05 n = 3; ** p<0.005 n = 6; triplicate samples, two-tailed unpaired t-test. Bars = +/- SEM. Prism, GraphPad).

As shown in Fig 6A and 6B, following oxaliplatin injection (3mg/kg ip), Sp4+/- mice failed to develop persistent cold hypersensitivity as measured at 4°C (48 hours– 14 days) and at 10°C at its maximal effect (72 hours) and 6 days, when compared to oxaliplatin-injected wt. mice. As shown in Fig 6C, oxaliplatin-induced mechanical hypersensitivity was initially observed up to 48 hours, but then was followed by a progressive reversal, attaining values similar to controls for Sp4+/- and wt. mice by days 14 and 21. Taken together, Sp4+/- mice were associated with a reduction in magnitude and reversal of persistent oxaliplatin-induced cold and mechanical hypersensitivity. Oxaliplatin’s action on peripheral nociceptors includes activation of nociceptive gene transcription, as evidenced by increased mRNA and functional channels encoding cold-sensing TRPA1 / TRPM8 and enhanced cold hypersensitivity [81–85]. In search for a possible explanation of why a decrease in Sp4 would result in a loss of oxaliplatin cold and mechanical hypersensitivity, as shown in Fig 6D, we found that both TRPA1 and TRPM8 mRNA expression was reduced in Sp4+/- DRG. Although future studies will be required to test this hypothesis, we speculate that persistent oxaliplatin-induced cold hypersensitivity is linked to Sp4-dependent transcription of TRPA1 and/or TRPM8.

Given the possibility that Sp4 may regulate other known genes implicated in mechanical hypersensitivity, we also probed a subset of genes previously reported to be expressed in DRG that have mechano-sensitive properties. As shown in Fig 6E, when expressed mRNA levels of ASIC3 [86], TRPV4 [87], Piezo1 [88], Piezo2 [89], TREK1 [90] and TMEM150c [63, 91] were examined, we were unable to observe a change in their mRNA expression as a result of a reduction of Sp4+/-.

## Discussion

Our findings suggest that the ability of certain hyperalgesic states to persist for days to weeks is dependent on the abundance and presumed activity of transcription factor Sp4. We propose that the expressed level of TRPV1 during an inflammatory state which is positively regulated by Sp4, is required for the persistence of inflammatory thermal hyperalgesia. Depending on the inflammatory and/or neuropathic condition, we also propose that Sp4 serves a broader role, beyond the regulation of TRPV1, in the development and persistence of painful mechanical and cold hypersensitive states.

### Sp4 is expressed in putative nociceptors

In support of Sp4 playing a critical role in the regulation of peripheral pain transduction, we examined the neurochemical characteristics of Sp4-positive lumbar DRG neurons using antisera to TRPV1 and IB4, established markers of primary afferent nociceptors, [80] as well as anti-NF200 and anti-peripherin antibodies [65]. Together, with our previous report of Sp4 mRNA and immune-reactive Sp4 protein expression in DRG [18], our histochemical characterization of Sp4+ DRG neurons (Fig 1) appears to unite two overlapping populations of small-medium sized DRG neurons under resting conditions: TRPV1+ thermally-responsive and IB4+ mechanically-responsive nociceptors [92–94]. Since small-medium sized TRPV1-positive and IB4-positive neurons with unmyelinated axons are considered to be nociceptive [1], our results suggest that Sp4 expression in DRG is preferentially expressed in a subset of primary afferent nociceptors.

Beyond our evidence that certain Sp4+ DRG neurons co-express TRPV1 (Fig 1G and 1H), we sought to link the consequence of a 50% reduction of Sp4 on measures of TRPV1 expression and functional activity *ex vivo*. We found a reduction in TRPV1 mRNA and in the magnitude of capsaicin-evoked calcium responses in Sp4+/- DRG neurons (Fig 2). These findings are consistent with our prior characterization of a dual promoter structure of the rat TRPV1 gene [17]. In our model, the promoter closest to the predominant RNA start site of TRPV1 transcription, promoter P2, reflects a Sp4-targeted binding sequence (GC box) and evidence of Sp4 binding by ChIP assay. We also proposed the existence of an upstream promoter P1, reflecting a TATA box-containing structure not associated with Sp4 binding [17, 18]. Although not yet fully characterized, it is likely that other factors contribute to the expression of TRPV1 in DRG neurons and may utilize the P1 and/or P2 sites within the dual promoter structure [95]. These include nuclear factors Runx1 and C/EBPbeta proposed to bind and activate a proximal TRPV1 promoter site in PC12 cells [95] and HDAC4, proposed to regulate TRPV1 expression in inflammatory hyperalgesia [96]. Together with our immunohistochemical observations (Fig 1), we propose that at least two subpopulations of TRPV1-expressing DRG neurons exist. One under the regulation of Sp4 and responsible for inflammatory thermal hyperalgesia (promoter P2) and another under the regulation of promoter P1.

### Inflammatory thermal hyperalgesia in Sp4+/- mice is not persistent

The activation of TRPV1 under conditions of inflammation is understood to drive the pathophysiology of thermal hyperalgesia, as measured by a decrease in thermal paw withdrawal latency and its absence under a null genetic phenotype (TRPV1-/-) [36, 37] [97–99]. We observed that although the CFA-treated Sp4+/- mice with a ~30% reduction of TRPV1 mRNA resulted in the early (2–6 hour) development of inflammatory thermal hyperalgesia (Fig 3C), thereafter, Sp4+/- mice underwent a spontaneous resolution of the pain behavior–despite ongoing evidence of inflammatory paw swelling (Fig 4). Investigating this further, we went on to compare these findings with that observed in mice with a fixed (50%) reduction in TRPV1 expression but presumably normal transcriptional activity in the active allele. As shown in Fig 3D, CFA TRPV1+/- heterozygous mice developed thermal hyperalgesia only at 2 hours, followed by its resolution to control values. Peripheral inflammation has been reported to lead to an increase in TRPV1 expression in a subpopulation of DRG neurons, including CGRP positive and IB4 positive rat DRG neurons [13, 100]. Therefore, under inflammatory conditions driving an increased demand for TRPV1, a reduction in TRPV1 transcriptional capacity at promoter P2 as a result of the Sp4+/- heterozygous state represents a plausible model to explain the development then loss of thermal hyperalgesia over a time frame of hours.

### Sp4+/- mice fail to develop persistent NGF-dependent mechanical hypersensitivity

Nerve Growth Factor (NGF), a neurotrophic factor and product of inflammation, has been shown to be essential for peripheral nociceptor development and to help maintain the nociceptor phenotype in adulthood–especially TRPV1 expression [11, 101–104]. Under conditions of peripheral inflammation including CFA, tissue and nerve injury, increased levels of NGF direct both acute and persistent hyperalgesic states in animals and humans [14, 15, 69, 105–107]. As shown in Fig 5, experimental models of intraplantar injection of NGF can induce short-term thermal hyperalgesia. Whereas both thermal and mechanical hyperalgesia initially develop rapidly, only NGF-induced mechanical hypersensitivity continued longer-term and can persist for weeks [47, 108, 109]. These observations have supported the notion that NGF drives not only acute nociceptor sensitization at the level of the peripheral transducing elements as exemplified by TRPV1 [71, 110–113], but also drives persistent hyperalgesic states through increased membrane expression [72, 114], translation of nociceptive proteins [115] and likely transcription [17].

As shown in Fig 5A, we observed a rapid development of NGF-induced mechanical hypersensitivity in Sp4+/- mice, but one reduced in magnitude that began to spontaneously resolve after 48 hours, returning to control—baseline values in 10 days. Although the mechanism(s) of a Sp4+/- associated blockade of NGF-induced mechanical hypersensitivity is unknown, it is plausible to speculate that it includes reduction of nociceptive components directly involved in mechanotransduction involving Sp4-dependent genes—beyond TRPV1. This may include TRPA1, given its association with mechanical hypersensitivity [116–118], ability to be upregulated by NGF [119] and expression in IB4-expressing DRG neurons [120]. A neuronal population we now observe to co-express Sp4 (Fig 1I). Moreover, Sp4+/- DRG is associated with reduced TRPA1 mRNA expression (Fig 6D). Future studies will be required to fully define the gene(s) responsible for Sp4-dependent mechanical hypersensitivity.

### Sp4+/- did not block the development of mechanical hypersensitivity in a model of spared nerve injury (SNI)

We studied a model of neuropathic pain (spared nerve injury—SNI) [48] and observed no difference in the time of onset, magnitude or duration of mechanical threshold testing between Sp4+/- and wt. mice (Fig 5C). Although models of nerve injury have been associated with an increase in NGF production, a driver for neuropathic pain behaviors, the disparity between CFA / NGF versus SNI in Sp4+/- mice may reflect the idea that SNI-induced mechanical hypersensitivity is primarily dependent on the activation of spinal microglia that in turn are proposed to induce and sustain mechanical hypersensitivity [49]. Conversely, microglial activation is not well correlated to inflammation (CFA-induced) decreases in mechanical threshold testing [121]. Overall, this suggests that a divergent set of SNI-dependent pathways and/or genes exist that are largely independent from Sp4 transcriptional regulation.

### Sp4+/- mice fail to develop persistent oxaliplatin-induced cold and mechanical hypersensitivity

Chemotherapy Induced Peripheral Neuropathy (CIPN) encompasses a wide range of clinical symptoms that include early post-treatment pain, paraesthesias, sensory ataxia, and is inclusive of oxaliplatin, cold and mechanical allodynia (for review, see [122, 123]). Despite the pain associated with platinum-based anticancer agents being widely recognized and certain underlying mechanisms elucidated [75–77], efforts to translate these findings into effective treatments in clinical practice have been disappointing [77, 82, 122–127]. As shown in Fig 6, Sp4+/- mice failed to develop persistent oxaliplatin-induced cold hypersensitivity (4°C; 10°C) and mechanical hypersensitivity. Since the peripheral sensory abnormalities associated with oxaliplatin are understood to be primarily a consequence of their binding to the DNA of sensory neurons [128], the reversal and subsequent loss of oxaliplatin-induced pain behaviors in Sp4+/- mice implies that exposure to platinum agents is not linked irreversibly to these pain behaviors.

Although we have suggested TRPA1 and TRPM8 as Sp4-dependent genes underlying the persistence of certain oxaliplatin-induced hypersensitivity, oxaliplatin-induced pain behaviors due to remodeling of non-TRP channels have also been reported [129]. Nevertheless, in a targeted analysis of other genes with properties of mechanical activation expressed in DRG, we failed to demonstrate that ASIC3, TRPV4, Piezo1, Piezo2, TREK1 or TMEM150c were significantly changed under conditions of Sp4+/- knockdown (Fig 6E). Therefore, it is unlikely that Sp4- dependent changes in the abundance of these genes explain the reduction and/or reversal of mechanical hypersensitivity observed in our experiments. Rather, one may speculate that Sp4-dependent loss of mechanical hypersensitivity is multifactorial. This may include contributions by the CNS given the limitation of our Sp4 knockdown mice [23–28] and reports that other regulatory features may be involved, such as changes in Sp4-dependent dendritic pruning–a form of synaptic plasticity [41]. Therefore, our findings suggest that ongoing gene transcription by Sp4, likely in nociceptors, play a critical role to sustain oxaliplatin-induced cold and mechanical hypersensitivity.

## Conclusions

Given its expression in a subset of small to medium sized DRG neurons with nociceptor markers, transcription factor Sp4 is well positioned to regulate nociceptor genes that engender critical roles in the control of pain transduction and the persistence of hypersensitive states. Consistent with this notion, a 50% reduction of Sp4 expression resulted in a profound loss of persistent hypersensitivity across multiple behavior models of pain in the time course of hours. Although our studies initially focused on the relationship between Sp4, TRPV1 and thermal hyperalgesia, our results more broadly suggest that a larger Sp4-dependent gene network or nociceptive transcriptome exists inclusive of TRPV1, TRPA1 and TRPM8. This implies that Sp4 is a critical control point for interrupting a network of genes underlying the persistence of painful hypersensitive states.

## Supporting information

S1 File Figs 1–6 data and statistics. (XLSX)
